# Supplementary material for: Exploring interactions between Beauveria and Metarhizium strains through co-inoculation and responses of perennial ryegrass in a one-year trial
Source: PeerJ. 2022 Mar 21;10:e12924. doi: 10.7717/peerj.12924 (PMC8944343; doi:10.7717/peerj.12924)

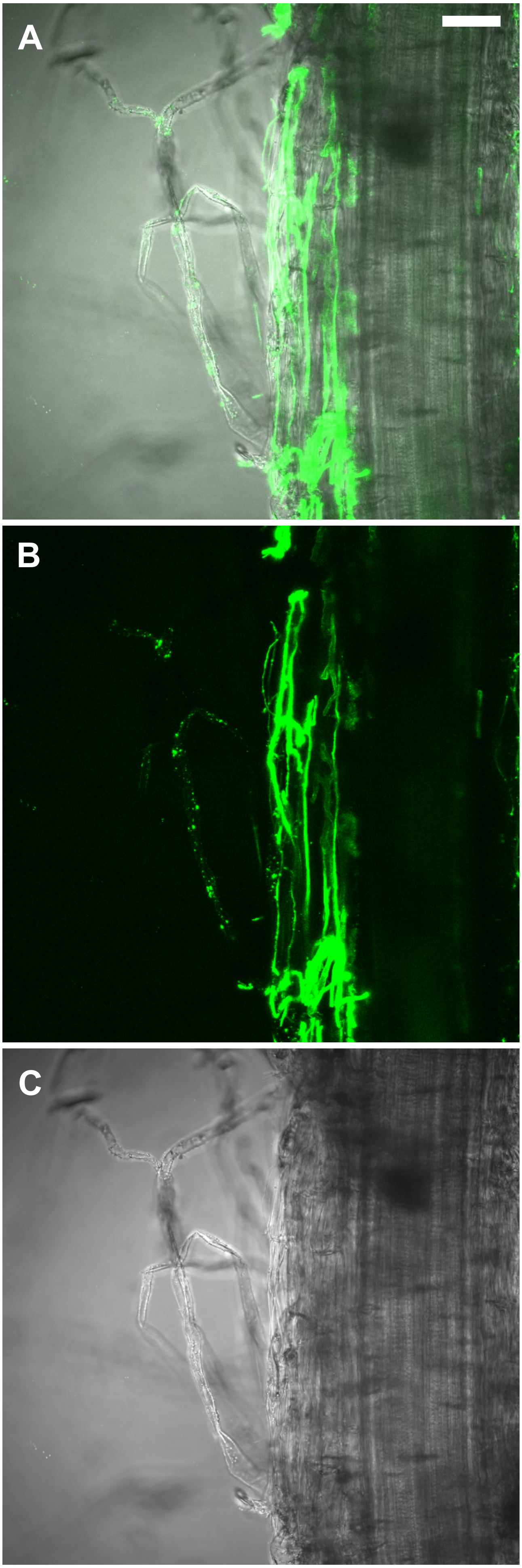


**Hyphal growth can extend into the rhizodermal cell layer**

A-C: Maximum projection of z-stacks, Overlay image (A), fluorescent channel (B), bright field image (C), magnified portion of the fluorescent image (D, one z-slice). Arrow indicates growth of hyphae into the rhizodermal cell layer. Bar size: 50 μm.

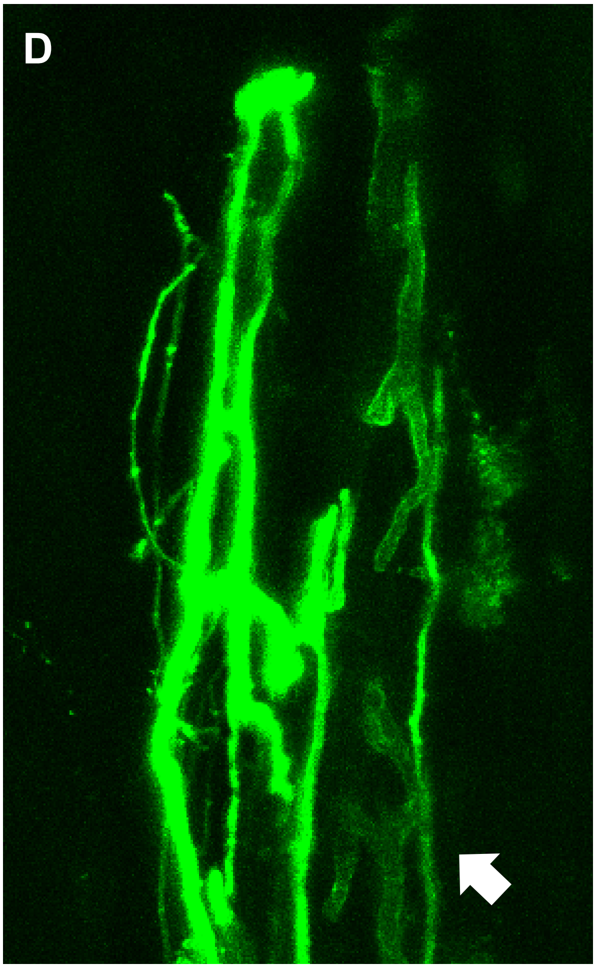

Supplement: Supplemental Information 1 — Confocal fluorescence images of a root section of a Lolium perenne plant 8 weeks after inoculation, stained with Wheat Germ Agglutinin-Alexafluor 488 (WGA-AF 488). Overlay of fluorescence image and bright field image (A), Fluorescence image (B), Bright field image (C). A magnified portion of one z-slide of image B is shown in (F). Scale bar represents 50 μm. [file peerj-10-12924-s001.pdf]
