# Supplementary material for: Exploring interactions between Beauveria and Metarhizium strains through co-inoculation and responses of perennial ryegrass in a one-year trial
Source: PeerJ. 2022 Mar 21;10:e12924. doi: 10.7717/peerj.12924 (PMC8944343; doi:10.7717/peerj.12924)
Supplement: Supplemental Information 3 — The Lolium perenne seeds correspond to the varieties commonly commercialized in Chile. [file peerj-10-12924-s003.docx]

|  | | | | |
| --- | --- | --- | --- | --- |
| **Seeds** | **Endophyte** | **Germination (%)** | **Bacterial growth (%)** | **Fungal growth (%)** |
| NUI | Variable | 82 | 35 | 0 |
| TOWER | Protek | 94 | 62 | 9 |
| RODEO | Edge | 92 | 79 | 1 |
| 24 SIETE | Edge | 92 | 84 | 0 |
| MATHILDE LE | - | 88 | 86 | 0 |
| STELLAR AR1 | AR1 | 89 | 92 | 0 |
| RODEO AR1 | AR1 | 94 | 100 | 0 |
| ANSA HAPPE | Happe | 95 | 100 | 1 |
