# Supplementary material for: Exploring interactions between Beauveria and Metarhizium strains through co-inoculation and responses of perennial ryegrass in a one-year trial
Source: PeerJ. 2022 Mar 21;10:e12924. doi: 10.7717/peerj.12924 (PMC8944343; doi:10.7717/peerj.12924)
Supplement: Supplemental Information 4 — Information obtained from Soil Laboratory, Austral University of Chile, 2020. [file peerj-10-12924-s004.docx]

| **Soil parameter Measure Unit Value** | | | |
| --- | --- | --- | --- |
| pH | in water | (1:2,5) | 60 |
| pH | CaCl2 (0,01M) | (1:2,5) | 5,4 |
| Organic material |  | (%) | 9,2 |
| N-Mineral | (N-NO3+NH4) | (mg/kg) | 19,6 |
| Phosphorus | Olsen | (mg/kg) | 7,4 |
| Potassium | interchangeable | (mg/kg) | 153 |
| Sodium | interchangeable | (cmol/kg) | 0,09 |
| Calcium | interchangeable | (cmol/kg) | 7,86 |
| Magnesium | interchangeable | (cmol+/kg) | 0,47 |
| Sum of bases | interchangeable | (cmol+/kg) | 8,81 |
| Aluminium | interchangeable | (cmol+/kg) | 0,03 |
| Effective cation exchange capacity |  | (cmol+/kg) | 8,84 |
| Aluminium saturation |  | (%) | 0,3 |
| Sulfur | available | (mg/kg) | 42,2 |
| Boron | available | (mg/kg) | 0,15 |
| Zinc | available | (mg/kg) | 1,88 |
| Iron | available | (mg/kg) | 83 |
| Copper | available | (mg/kg) | 2,15 |
| Manganese | available | (mg/kg) | 16,1 |
| Aluminium | extractable | (mg/kg) | 898 |
| Information obtained from Soil Laboratory, Austral University of Chile, 2020. | | | |
